# IL-27 Mediates PD-L1 Expression and Release by Human Mesothelioma Cells

**DOI:** 10.3390/cancers13164011

**Published:** 2021-08-09

**Authors:** Grazia Carbotti, Beatrice Dozin, Stefania Martini, Chiara Giordano, Francesca Scordamaglia, Michela Croce, Gilberto Filaci, Silvano Ferrini, Marina Fabbi

**Affiliations:** 1 Biotherapies Unit, IRCCS Ospedale Policlinico San Martino, Largo R. Benzi 10, 16132 Genoa, Italy; graziacarbotti@gmail.com (G.C.); chiara_giordano@live.it (C.G.); michela.croce@hsanmartino.it (M.C.); gfilaci@unige.it (G.F.); 2 Clinical Epidemiology Unit, IRCCS Ospedale Policlinico San Martino, Largo R. Benzi 10, 16132 Genoa, Italy; dozinb@libero.it; 3 Immunology Unit, IRCCS Ospedale Policlinico San Martino, Largo R. Benzi 10, 16132 Genoa, Italy; stefania.martini@hsanmartino.it; 4 Department of Pneumology, Azienda Ospedaliera Villa Scassi, Corso O. Scassi 1, 16149 Genoa, Italy; scorda78@libero.it; 5 Centre of Excellence for Biomedical Research and Department of Internal Medicine, University of Genoa, Via De Toni 14, 16132 Genoa, Italy; 6 Department of Experimental Medicine, University of Genoa, Via L.B. Albertis 2, 16132 Genoa, Italy

**Keywords:** mesothelioma, IL-27, PD-L1, IL-6, STAT1/3, pleural effusion, microenvironment, overall survival

## Abstract

**Simple Summary:**

Malignant mesothelioma (MM), a rare and aggressive tumor, is related to asbestos exposure, which mediates a chronic inflammatory process involving the cytokine IL-6. Recent studies indicate that the PD-1/PD-L1 immune-suppressive axis is a clinically relevant target for therapy. The expression of PD-L1 in tumor cells is generally a cytokine-mediated effect. Here we show that the IL-6-related cytokine IL-27 is able to enhance PD-L1 expression and soluble (s)PD-L1 release by cultured MM cells, whereas IL-6 is ineffective. In agreement with previous findings, we found high IL-6 levels in pleural exudates from 77 MM patients which correlated with worse survival. More importantly, we also found sPD-L1 and IL-27 in the same samples. sPD-L1 and IL-27 levels showed a moderate albeit significant correlation and association with worse survival, which suggested a potential effect of IL-27 on PD-L1-mediated immune resistance in MM.

**Abstract:**

Malignant mesothelioma (MM) is a rare tumor with an unfavorable prognosis. MM genesis involves asbestos-mediated local inflammation, supported by several cytokines, including IL-6. Recent data showed that targeting PD-1/PD-L1 is an effective therapy in MM. Here, we investigated the effects of IL-6 trans-signaling and the IL-6-related cytokine IL-27 on human MM cells in vitro by Western blot analysis of STAT1/3 phosphorylation. The effects on PD-L1 expression were tested by qRT-PCR and flow-cytometry and the release of soluble (s)PD-L1 by ELISA. We also measured the concentrations of sPD-L1 and, by multiplexed immunoassay, IL-6 and IL-27 in pleural fluids obtained from 77 patients in relation to survival. IL-27 predominantly mediates STAT1 phosphorylation and increases PD-L1 gene and surface protein expression and sPD-L1 release by human MM cells in vitro. IL-6 has limited activity, whereas a sIL-6R/IL-6 chimeric protein mediates trans-signaling predominantly via STAT3 phosphorylation but has no effect on PD-L1 expression and release. IL-6, IL-27, and sPD-L1 are present in pleural fluids and show a negative correlation with overall survival, but only IL-27 shows a moderate albeit significant correlation with sPD-L1 levels. Altogether these data suggest a potential role of IL-27 in PD-L1-driven immune resistance in MM.

## 1. Introduction

Malignant mesothelioma (MM) is a rare tumor arising from the mesothelial cells underlining the pleural, peritoneal, or, less frequently, pericardial cavities. In spite of aggressive treatments including surgery, radiotherapy, and/or chemotherapy, the prognosis of MM remains poor, with a median overall survival of about 15 months [1]. Immunotherapy with the anti-CTLA-4 mAb tremelimumab has shown no impact on survival when administered as a second- or third-line therapy in MM patients [2]. However, immunotherapy with anti-PD-1 or anti-PD-L1 mAbs alone or in combination with anti-CTLA-4 mAbs showed promising activity in relapsing MM patients [3,4,5,6,7,8,9]. More importantly, a recent phase III study showed that the combination of nivolumab and ipilimumab improved overall survival versus standard-of-care chemotherapy in unresectable MM [10]. These data support a role for the PD-1/PD-L1 interaction as a clinically relevant immune-suppressive mechanism in MM. Indeed, high PD-L1 expression in MM tissues correlates with a more severe prognosis [11,12].

MM genesis is strictly linked to asbestos fiber exposure, which causes necrotic cell death and the release of the high mobility group box protein-1 (HMGB1) [13]. In turn, HMGB1 acts as a danger signal triggering the NALP3 inflammasome, which induces a chronic inflammatory response via secretion of TNF and IL-1β by macrophages [14,15]. Several other cytokines and chemokines are then involved in this chronic inflammatory process [16,17]. In particular, auto/paracrine IL-6 has been reported to play a major role in MM [18] as its concentration is elevated in MM sera and pleural fluids [19,20].

IL-6 signals through a membrane heterodimer receptor consisting of the IL-6R and GP130 chains. Besides this classical signaling, IL-6 can also bind to a soluble (s) form of IL-6R, and this complex can mediate trans-signaling in cells expressing the GP130 chain, irrespective of surface IL-6R expression [21]. A previous study supported a role for IL-6 trans-signaling in promoting MM cell proliferation and VEGF production in vitro [22]. IL-6 was also reported to induce PD-L1 expression in myeloid cells in a murine glioblastoma model [23] and in human myeloma cells [24].

IL-6 is part of a large family of cytokines, including IL-27, which share the usage of a common GP130 receptor chain in their receptor complexes [25,26]. Whereas IL-6 has mainly pro-tumor activities, IL-27 may have a dual biological role in different cancers [27]. IL-27 is also a member of the IL-12 family of heterodimer cytokines [28,29]. It consists of p28 and EBI3 chains and binds to a heterodimer receptor consisting of GP130 and WSX1 [30].

IL-27 induces a set of proteins broadly overlapping with those induced by IFN-γ [31] and shares several anti-tumor functions in common with IFNs, including anti-angiogenic activities [32], induction of HLA-class I molecule expression [33,34,35], and anti-proliferative activity on cancer cells [36,37]. In addition, similar to IFN-γ [38,39], it also mediates the expression of PD-L1 and the immune-suppressive enzyme IDO in cancer cells [40]. Therefore, both IFN-γ and IL-27 induce immune-regulatory circuits, which may limit anti-tumor immunity. In a recent report, we found that high IFN-γ levels in pleural exudate correlate with better prognosis in a cohort of MM patients presenting mostly a stage I-II tumor [41]. However, IFN-γ levels were lower and showed no association with prognosis in a different cohort of MM patients with advanced-stage disease.

In the present study, we first addressed the effects of IL-27 on MM cell lines in vitro. In addition, we tested the effects of IL-6 and IL-6 trans-signaling through a sIL-6R/IL-6 chimeric protein, mimicking the sIL-6R/IL-6 complex. Interestingly, IL-27 increased PD-L1 surface expression and soluble (s)PD-L1 secretion in MM cell lines, supporting a potential role of IL-27 in inducing PD-1/PD-L1 immune-suppressive circuits. We thus measured the levels of immune-reactive IL-6, IL-27, and sPD-L1 in MM pleural fluids and assessed whether these factors correlated with survival in a cohort of 77 MM patients.

## 2. Results

### 2.1. IL-27 and a sIL-6R/IL-6 Chimeric Protein Mediated Signal Transduction in Human MM Cells

**Figure 1:**
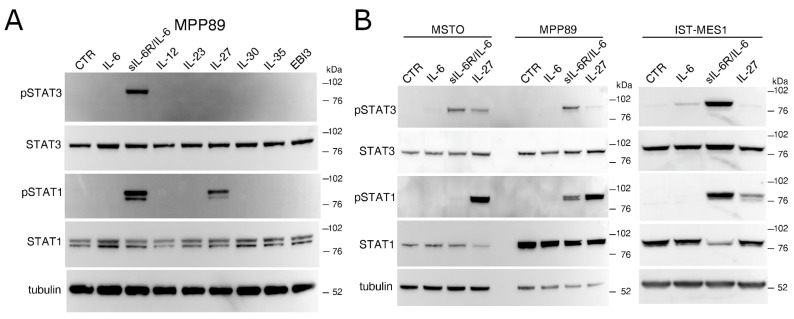
IL-27 predominantly mediates STAT1 phosphorylation in MM cell lines. Western blot analysis of tyrosine phosphorylated (P)-STAT1, P-STAT3, total STAT1 and total STAT3 proteins (**A**) in MPP89 cells stimulated for 20 min with medium (CTR) or the indicated cytokines of the IL-12 family and (**B**) in MSTO, MPP89, and IST-MES1 cells stimulated for 20 min with medium (CTR), IL-6, sIL-6R/IL-6 chimera, and IL-27. Total STATs and α-tubulin were used as loading controls.

To assess the responsiveness of MM cells to IL-27 and other related cytokines of the IL-12 family, we treated MPP89 cells and performed Western blot analysis of tyrosine-phosphorylated STAT1 and STAT3 proteins (Figure 1A). We tested a panel of cytokines sharing one of the two chains (p28 or EBI3) with IL-27, i.e., EBI3, IL-12, IL-23, IL-30, and IL-35. In addition, we also tested IL-6 and a sIL-6R/IL-6 chimeric protein, capable of mediating IL-6 trans-signaling.

As shown in Figure 1A, the MMP89 cell line responded to sIL-6R/IL-6 through STAT3 and STAT1 phosphorylation, while IL-27 predominantly induced STAT1 activation. By contrast, IL-6 and the IL-27-related cytokines EBI3, IL-12, IL-23, IL-30, and IL-35 failed to trigger STAT1/3 signal transduction.

Consistent results were obtained with the other two MM cell lines, MSTO and IST-MES1, of which only one (IST-MES1) also weakly responded to IL-6, whereas all cell lines phosphorylated STAT3 and to a lesser extent STAT1 in response to sIL-6R/IL-6 stimulation (Figure 1B). Overall, IL-27 predominantly induced STAT1 phosphorylation and a weaker phosphorylation of STAT3 as compared to the sIL-6R/IL-6 chimeric protein. As expected, stimulation with IFN-γ predominantly triggered STAT1 phosphorylation in MM cell lines similar to IL-27 (Supplementary Materials Figure S1).

### 2.2. IL-27, but Not sIL-6R/IL-6 Chimeric Protein, Mediates Expression of Surface PD-L1 Molecule and Release of Soluble PD-L1 by MM Cells

Results of clinical studies with anti-PD-1/PD-L1 immune checkpoint blockers supported the relevance of this immune regulatory pathway in MM [10]. Since IL-27 can induce PD-L1 expression in different tumor cell types [40], we assessed IL-27’s capability of upregulating PD-L1 expression in human MM cell lines as compared to the sIL-6R/IL-6 chimera.

**Figure 2:**
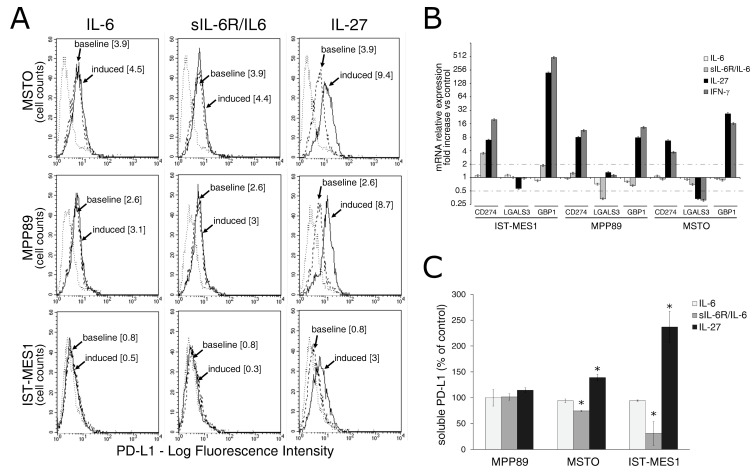
IL-27 upregulates PD-L1 molecule expression and release by MM cells. (**A**) Flow cytometry analysis of membrane PD-L1 expression in MSTO, MPP89, and IST-MES1 MM cell lines cultured with medium alone (baseline), IL-6, sIL-6R/IL-6 chimera, or IL-27 (induced). Dotted line shows isotype-matched Ig control. Numbers in brackets represent Median Fluorescence Intensity (MFI) values calculated as median anti-PD-L1 mAb minus median Ig control. Data are representative of two independent experiments yielding similar results. (**B**) qRT-PCR analysis of *CD274* (PD-L1) mRNA expression in the same three MM cell lines stimulated with IL-6, sIL-6R/IL-6 chimera, IL-27, or IFN-γ relative to untreated cells. Data are calculated with the ΔΔCT method and expressed as fold change versus untreated control. Error bars represent SD of triplicates. *LGALS3* (Galectin 3) and *GBP1* (Guanylate Binding Protein 1) mRNA levels are shown, respectively, as negative and positive controls of IL-27 activity. (**C**) Evaluation by ELISA of soluble (s)PD-L1 release in the conditioned media of the indicated MM cells treated with IL-6, sIL-6R/IL-6 chimera, or IL-27. Data are expressed as a percent of untreated control and are the mean of two independent experiments, run in duplicates. Error bars represent the minimum and maximum values (* *p* < 0.001, Student’s *t*-test versus untreated control).

Cytofluorimetric analyses indicate that IL-27 induced an average three-fold increase in surface PD-L1 expression relative to constitutive expression, in the three MM cell lines tested. By contrast, IL-6 and sIL-6R/IL-6 chimera failed to modulate surface PD-L1 expression (Figure 2A). Notably, two out of three cell lines showed constitutive expression of surface PD-L1 relative to isotype Ig control. In a second set of experiments, we compared the effect of IL-27 with that of IFN-γ, which is a known inducer of PD-L1 [39,42,43], and found that both stimuli mediated PD-L1 cell surface upregulation (Supplementary Materials Figure S2A).

Consistently, expression of *CD274* (PD-L1) mRNA was enhanced by IL-27 or IFN-γ treatment, whereas the IL-6 and sIL-6R/IL-6 induced no or minor changes in the MM cell lines tested (Figure 2B). As a control of IL-27 or IFN-γ activity, we assessed the expression levels of the *GBP1* gene, which is upregulated by both cytokines [44].

Recent data indicated that IFN-γ is capable of mediating not only PD-L1 surface expression but also soluble (s)PD-L1 release by human MM cells [45]. We, therefore, assessed whether IL-27 may have similar effects. As shown in Figure 2C, IL-27 triggered sPD-L1 release from two out of three MM cell lines. By contrast, IL-6 or sIL-6R/IL-6 chimera failed to induce sPD-L1 release.

Since IL-27 is known to upregulate surface HLA Class I expression in different types of human cancer cells [31,33,34,35], we assessed whether this effect also occurred in MM cells. As shown in Supplementary Materials Figure S2B, human MM cells constitutively expressed surface HLA Class I heavy chains in complex with β2-microglobulin, as detected by the W6/32 mAb, in agreement with previous findings [46,47,48]. IL-27 further enhanced cell surface expression of HLA Class I/β2-microglobulin complexes, while sIL-6R/IL-6 chimeric protein was ineffective in this respect.

### 2.3. Immuno-Reactive IL-27 Is Detectable in MM Pleural Exudates and Correlates with Soluble PD-L1 Expression

**Figure 3:**
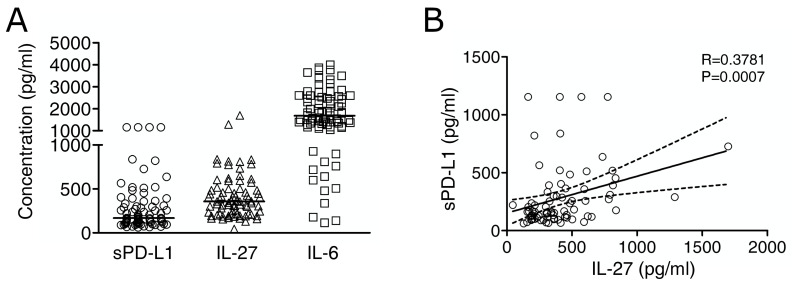
IL-27 is detectable in MM pleural exudates and correlates with sPD-L1 expression. (**A**) Concentration (pg/mL) of sPD-L1, IL-27, and IL-6 in pleural effusion. Bars indicate median values. (**B**) Correlation between IL-27 and sPD-L1 levels in MM pleural fluids. Spearman’s rank correlation coefficient (R) and *p*-value (P) are indicated. Lines represent the best fit linear regression analysis with the 95% confidence interval.

To gain information on the potential role of IL-27 and IL-6 in MM in vivo and their possible relationship with sPD-L1, we assessed the levels of these molecules by Luminex or ELISA assays in MM pleural fluids. We detected sPD-L1 (median value 166.2 pg/mL, minimum 61.5, maximum 1154.1), IL-27 (median 357.9 pg/mL, min. 44.9, max. 1698) and IL-6 (median 1683 pg/mL, min. 113.9, max. 4003) at variable levels in a cohort of 77 MM cases (Figure 3A).

Interestingly, IL-27 levels showed a moderate (R = 0.3781) albeit highly significant (*p* = 0.0007) correlation with sPD-L1 levels, suggestive of a potential role for IL-27 in PD-L1 upregulation in vivo (Figure 3B).

This observation was confirmed using a binomial logistic regression (Table 1).

As can be seen, patients with high IL-27 concentrations were almost five times more likely to exhibit high concentrations of sPD-L1 as compared to patients with low IL-27 concentrations (OR = 4.86, 95% CI 1.86–12.79, *p* = 0.001). By contrast, both low and high concentrations of IL-6 correlated to the same extent with high concentrations of sPD-L1 (OR = 0.95, 95% CI 0.39–2.32, *p* = 0.910).

Altogether, these data indicated a potential role for IL-27 in PD-L1-mediated immune resistance in MM. Since PD-L1 expression in mesothelioma cells is induced by IFN-γ in vitro, we also investigated the possible correlation between sPD-L1 and IFN-γ concentrations in pleural fluids in our patients’ cohort. To this end, we first re-assessed IFN-γ in the same samples by a high-sensitivity ELISA kit, as our previous results showed that IFN-γ levels were below the threshold sensitivity of the Luminex assay [41]. Here we confirmed the presence of overall low levels of IFN-γ (median value 0.884 pg/mL, min. 0.001, max. 31.47), which showed a moderate but significant correlation with sPD-L1 concentrations and a weaker correlation with IL-27 levels (Supplementary Materials Figure S3).

### 2.4. Overall Survival According to PD-L1, IL-27, and IL-6 Concentrations in the Pleural Effusion

After the withdrawal of the pleural effusion sample, the 77 patients included in the study were followed for a median period of 42.2 months (95% CI 21.6–62.9). During this time frame, 66 of them (87.0%) died. Median overall survival (OS) was 10.4 months (95% CI 8.0–12.8).

For all three factors analyzed, a concentration below the median value favored OS. This effect was particularly significant for sPD-L1 and IL-6 and somehow less pronounced for IL-27.

**Figure 4:**
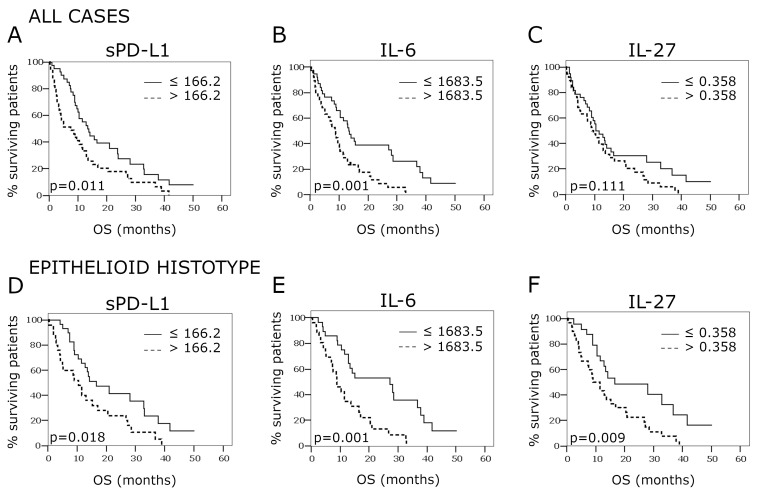
Kaplan–Meier curves assessing overall survival according to sPD-L1, IL-27, and IL-6 concentrations in the pleural effusion, as dichotomized around their respective median value. Analyses with respect to all cases (*n* = 77) (panels **A**–**C**) and the subgroup with the epithelioid histotype *(n* = 55) (panels **D**–**F**) are shown.

Specifically, median OS was 13.4 months (95% CI 9.4–17.5) for low sPD-L1 concentrations as compared to 7.5 months (95% CI 1.7–13.3) for high sPD-L1 concentrations (*p* = 0.011) (Figure 4, panel A). Similarly, survival was significantly better in patients with low IL-6 levels as compared to patients with high IL-6 levels (median OS 13.4 months (95% CI 11.3–15.5) vs. 8.6 months (95% CI 6.3–10.9, *p* = 0.001) (Figure 4, panel B). By contrast, IL-27 concentration did not significantly affect OS, the median values being 10.5 months (95% CI 6.4–14.6) and 9.1 months (95% CI 6.4–11.8) for low and high concentrations, respectively (*p* = 0.111) (Figure 4, panel C).

Most of the patients (*n* = 55) presented epithelioid mesothelioma. In this subgroup, the risk of death was reduced by almost 70% as compared to that of patients with other histotypes (HR = 0.31, 95% CI 0.18–5.4, *p* < 0.001; not shown). As in the whole cohort, OS was favored in this subgroup in the presence of low levels of sPD-L1 and IL-6 in the pleural effusion as compared to high levels: sPD-L1, 16.6 months (95% CI 7.0–26.2) vs. 10.5 months (95% CI 5.8–15.2), *p* = 0.018, Figure 4, panel D; IL-6, 27.2 months (95% CI 9.2–45.1) vs. 8.9 months (95% CI 5.3–12.5), *p* = 0.001, Figure 4, panel E. Moreover, IL-27 at low concentration also improved survival in the epithelioid subgroup with respect to high concentration (16.6 months (95% CI 0.7–33.5) vs. 9.1 months (95% CI 5.1–13.1), *p* = 0.009, Figure 4, panel F).

Finally, IFN-γ concentration measured by high-sensitivity ELISA did not significantly affect OS, the median OS values being 10.1 months (95% CI 6.4–13.8) and 10.4 months (95% CI 7.3–13.7) for low and high concentrations, respectively (*p* = 0.858). The same was true in the subgroup of patients presenting an epithelioid subtype (respective median OS were 11.5 and 13.4 months for low and high IFN-γ concentrations, *p* = 0.928) (Supplementary Materials Figure S3). Despite the lack of effect of IFN-γ on survival, we found that sPD-L1 concentrations significantly correlated with IFN-γ concentrations, as determined by a binomial logistic regression. Patients with high levels of IFN-γ were more than five times more likely to exhibit high levels of sPD-L1 as compared to patients with low IFN-γ levels (OR = 5.16, 95% CI 1.62–16.39, *p* = 0.005).

## 3. Discussion

It is well known that pro-inflammatory cytokines play a crucial role in human MM pathogenesis, progression, angiogenesis, resistance to therapy, and immune escape mechanisms [13,14,15,16,17,18]. In particular, cytokines, such as IFN-γ, released during T cell responses, upregulate the expression of PD-L1 on the tumor cell surface, thus inhibiting further activation of CTLs responses. This phenomenon has been reported as “adaptive immune resistance” [38,39,49,50] and is a major mechanism of immune escape in several tumors, as evidenced by the successful results of anti-PD-1/PD-L1 antibodies in cancer immunotherapy [38,49].

Recent reports indicated that IFN-γ upregulates membrane PD-L1 expression and soluble (s)PD-L1 release in human mesothelioma cells in vitro [43]. Therefore, adaptive immune resistance could represent a major mechanism of tumor escape and a target for therapy in human MM. Indeed, a recent randomized trial evaluating the combination of anti-PD-1 and anti-CTLA4 as first-line immunotherapy supports a role for the PD-1/PD-L1 interaction as a clinically relevant immune regulatory mechanism in MM [10]. Furthermore, sPD-L1 is found in the pleural effusion of mesothelioma patients in relationship with the PD-L1 expression at the level of the tumoral tissue [45].

Since we recently found that IFN-γ levels were very low in pleural exudates of advanced-stage MM [41], we wondered whether IL-27 could be involved in PD-L1 upregulation and release in advanced MM. Indeed, in a previous study, we showed that IL-27, a cytokine produced by macrophages or DCs, may provide an alternative stimulus for PD-L1 expression in different types of human cancer cells [40]. In the present study, we first demonstrated that MM cells responded to IL-27 by inducing STAT1 phosphorylation and, to a lesser extent, STAT3 activation in vitro, whereas other members of the IL-12-cytokine family were ineffective. Regarding the biological effects of IL-27 signaling on MM cells, we found that it upregulated the surface expression of HLA Class I molecules. However, since HLA Class I is already abundant on MM cells, such an effect could have a minor impact on MM immune recognition by CTLs. On the other hand, similar to IFN-γ [45], IL-27 increased surface PD-L1 expression, *CD274* (PD-L1) mRNA levels, and sPD-L1 release in MM cells, suggesting that it may promote a negative effect on T cell responses. Besides the induction of PD-L1 expression in tumor cells, IL-27 may have additional immune-suppressive activities through the induction of IL-10-producing or PD-L1-expressing regulatory-type CD4+ T cells, such as described in autoimmunity and GVHD models [51,52,53,54,55]. Whether these activities are relevant to immune regulation and disease progression in MM remains to be determined.

In addition to IL-27, we also studied IL-6, another cytokine involved in immune regulation, which may play a major role in MM oncogenesis and progression [18]. We here showed that IL-6 failed to trigger the signaling cascade in MM cells unless complexed with soluble (s)IL-6R. Indeed, a chimeric protein mimicking the sIL-6R/IL-6 complex mediated trans-signaling mainly through STAT3 phosphorylation and a weaker STAT1 activation. These data are consistent with a previous report showing that IL-6 is able to mediate signaling in human MM cells only in the presence of sIL-6R, as MM cells lack surface expression of the IL-6R chain [22]. Although sIL-6R/IL-6 complexes mediated IL-6 trans-signaling in MM cells in vitro, this signaling did not result in increased PD-L1 expression in the MM cell panel tested.

We then verified the potential impact of IL-27 in the MM microenvironment by cytokine quantification in pleural fluids from a cohort of 77 MM patients. IL-27 was well detected within the standard curve range of values in all samples, indicating that it may play a role in vivo in MM. Moreover, high IL-27 levels were significantly associated with worse survival in the epithelioid MM subgroup of cases.

We also found that sPD-L1 was present in the same pleural exudates, in agreement with a previous report in a different MM cohort [45]. In addition, high sPD-L1 levels were significantly associated with worse survival in our cohort. Importantly, we found a moderate, albeit highly significant, correlation between IL-27 and sPD-L1 levels, which suggested a potential role for IL-27 in the activation of PD-L1/PD-1-mediated immune-regulatory pathway in advanced stage MM. In addition to IL-27, IFN-γ levels also showed a similar correlation with sPD-L1 in pleural exudates, suggesting that both IL-27 and IFN-γ may contribute to PD-L1 expression in the mesothelioma microenvironment. In addition, the low but significant correlation between IL-27 and IFN-γ concentration suggests a potential interplay between the two cytokines in a subset of patients. Previous data indicated that sPD-L1 may derive either from metalloprotease-mediated shedding of cell surface PD-L1 molecules [56,57] or from *CD274* mRNA alternative splicing, which generates a dimeric form of PD-L1 [58]. Both sPD-L1 forms were capable of binding surface PD-1 on T cells and mediated immune suppression [59,60]. Soluble PD-L1 may also interfere with immunotherapy with PD-L1-blocking mAbs as it competes with membrane PD-L1 [61,62,63] and impairs the anti-PD-L1-mediated antibody-dependent cellular cytotoxicity (ADCC) of MM cells. In this context, it was reported that MM cells are sensitive to anti-PD-L1 mAb-mediated ADCC [64,65].

Several reports indicated that IL-27 may play a dual role in cancer biology and immunotherapy [27,29,66]. Overall, the correlation between high IL-27 levels and worse survival in the epithelioid MM subgroup together with IL-27 ability to drive upregulation of membrane and sPD-L1 expression in vitro and the correlation between sPD-L1 and IL-27 in vivo support a pro-tumor, immune-suppressive role for IL-27 in advanced MM.

In agreement with previous data [18], we found that IL-6 was present at high concentrations in the pleural effusion of MM and correlated with a worse prognosis. More importantly, we found that high levels of sIL-6R were present in the same fluids (not shown), supporting the role of sIL-6R/IL-6 complexes as mediators of IL-6 trans-signaling in vivo in human MM.

In spite of the common negative impact of IL-6 and IL-27 levels on survival, the concentrations of the two cytokines varied independently. The lack of correlation may be related to the only partially overlapping cellular sources of IL-6 and IL-27. Indeed, IL-27 is mainly produced by myelomonocytic cells [27,67], while IL-6 can be secreted by several other cell types, including T lymphocytes and tumor cells [68].

In conclusion, our data indicate that IL-27 is present at meaningful concentrations in MM pleural exudates and may play an immune regulatory role in advanced-stage MM, possibly through the induction of PD-L1 expression.

## 4. Materials and Methods

### 4.1. Ethics Approval and Consent to Participate

This study was conducted in accordance with the ethical standards and according to the Declaration of Helsinki and National and International guidelines and has been approved by the authors’ Institutional Review Board (Study n. N9-13, ASL3. Regional platform id 4975). Pleural fluids were collected during surgical procedures from patients who gave written informed consent and were used following the Institutional Review Board approval.

### 4.2. Cells and Treatments

The human MM cell lines MPP89 and IST-MES1 [48] and MSTO were obtained from Interlab Cell Line Collection (ICLC, Ospedale Policlinico San Martino, Genoa, Italy) and were never cultured for more than 4 months after thawing an aliquot of the original stock. Cells were grown in RPMI 1640, with L-glutamine, 10% FCS, and antibiotics (Lonza) at 37 °C in 5% carbon dioxide. The human cytokines used were: IL-27 (2526-IL-010), IL-6 (206-IL-010), recombinant human IL-6R/IL-6 chimera (8954-SR-025), IL-12 (219-IL-005), and IL-23 (1290-IL-010) from R&D Systems, IFN-γ (PeproTech, 300–02), IL-35 (Enzo Life Sciences, ALX-522-140-C010), IL-30 (Abnova, H00246778-P01), and EBI3 (Novus Biologicals, P4568). Treatments with cytokines were set on the basis of preliminary titration experiments and previous reports [31,35,40,44]. Briefly, for immunofluorescence, RT-PCR, and ELISA of conditioned media, MM cells were seeded in 24-well plates at 5 × 10^4^ cells/well, and cytokines were added for a 48-h treatment: IL-27 at 100 ng/mL, IFN-γ at 1000 IU/mL, IL-6 at 50 ng/mL, or recombinant human IL-6R/IL-6 chimera at 50 ng/mL. For Western blot analysis of tyrosine-phosphorylated STAT proteins, 10^5^ cells were incubated for 20 min at 37 °C in 0.5 mL of medium containing IL-27 (50 ng/mL), IL-6 (20 ng/mL), sIL-6R/IL-6 (40 ng/mL), or IFN-γ (1000 IU/mL). Cells were then recovered by centrifugation and immediately processed.

### 4.3. Western Blot

Cells were lysed with 20 mM Tris-HCl pH 7.4, 1 mM EDTA, 150 mM NaCl, 1% Brij97, 2 mM Na Orthovanadate, and protease inhibitors (Roche Diagnostics, Complete Mini 04693124001). Proteins were resolved by 10% SDS-PAGE under reducing conditions, and Western blotting was carried out according to standard techniques, with rabbit anti-phospho-STAT1 (pY701) and anti-STAT1 anti-sera (Cell Signaling Technology, 9167 and 9172, respectively), mouse anti-phospho-STAT3 (pY705) and anti-STAT3 mAbs (BD Transduction Laboratories, 612,356 and 610,190, respectively) and α-tubulin mAb (Sigma-Aldrich, T6074).

Immunoreactive proteins were detected by ECL Prime (GE Healthcare, RPN2232) and a chemiluminescence gel documentation and analysis system (MINI HD, UVITEC, Cambridge, UK). Densitometric analyses of relevant bands and whole Western blot images are shown in the Supplementary Materials Figure S4.

### 4.4. Immunofluorescence and Flow Cytometry

Immunofluorescence with anti-PD-L1 PE or Isotype Control PE (eBioscience Bender, BMS-125983-41 and BMS-124724-41, respectively) was performed according to the manufacturer’s instructions. Indirect immunofluorescence with anti-HLA Class I W6/32 mAb (ATCC) and FITC-labeled goat anti-mouse (Jackson Immunoresearch, 115-096-068) was performed on 10^5^ cells/sample following standard procedures. Samples were analyzed by a FACScan (Becton & Dickinson) flow cytometer and the Cell Quest software, with gating on viable cells and acquisition of 10^4^ gated events.

### 4.5. RT-PCR Analysis

Total RNA was isolated by the NucleoSpin RNA kit (Macherey-Nagel, 740955.250) and reverse-transcribed using the SuperScript III Reverse Transcriptase (Invitrogen, 18064-071). Amplification was carried out using the iQTM SYBR® Green Supermix system (Bio-Rad Laboratories, 170–8882) by the Mastercycler® ep realplex4 instrument (Eppendorf International). Expression levels of mRNAs relative to untreated control were calculated by the ΔΔCT method. The primers used are listed in Supplementary Materials Table S1.

### 4.6. Cytokine Dosage and ELISA Tests

The concentrations of IL-27 and IL-6 in the MM pleural effusions were assessed with a bead-based multiplexed immunoassay and Luminex technology (MILLIPLEX MAP Human TH17 Magnetic Bead Panel, customized HTH17MAG-8K, Merck Millipore, Darmstadt, Germany), according to the manufacturer’s instructions. Samples were tested both at 1:2 and 1:10 dilutions in Assay Buffer.

The levels of soluble PD-L1 protein were measured by ELISA using the Human PD-L1 [28–8] SimpleStep ELISA Kit (Abcam, ab214565), following datasheet instructions. Both pleural effusions and conditioned media were centrifuged in a microfuge to eliminate debris and used undiluted. Results were processed by the BioTek EL808 absorbance microplate reader and GEN5 Software version 2.03.01.

### 4.7. Patients

A cohort of 77 MM patients was included in the study with the purpose of assessing survival. See Supplementary Materials Table S2 for clinical characteristics of cases. The cohort was obtained from the “Alessandria Biobank-Centro Raccolta Materiali Biologici”, Department of Integrated Activities Research and Innovation, Azienda Ospedaliera SS. Antonio e Biagio e Cesare Arrigo, Alessandria, Italy. These patients were subjected to diagnostic thoracentesis and thoracoscopy between July 2012 and July 2019. During these processes, samples of pleural effusion were withdrawn, debris and cells were removed by centrifugation at 1500× *g,* and fluids were cryopreserved in aliquots at −80 °C. The collection was approved by the local Ethical Committee, and all patients signed an informed consent.

### 4.8. Statistics

The concentrations of PD-L1, IL-6, and IL-27 reported as continuous variables were dichotomized on the basis of their median value. The association between PD-L1 concentration and IL-27 or IL-6 concentration was assessed using binomial logistic regressions. Odds ratio (OR) estimates, with 95% confidence intervals, were computed as a relative index of these associations. Overall survival (OS), as estimated from the date of pleural effusion withdrawal to the date of last contact or death from any cause, was analyzed using the Kaplan–Meier method, and differences between groups were assessed by the log-rank test.

## 5. Conclusions

In this report, we showed that the IL-6-related cytokine IL-27 mediates STAT1 signaling and PD-L1 gene and surface protein expression and sPD-L1 release by human MM cells in vitro. Conversely, IL-6 predominantly mediates STAT3 signaling but has no effect on PD-L1 expression. In addition, both IL-27 and sPD-L1 were present in pleural fluids from 77 MM patients and showed a relationship with worse overall survival. Finally, IL-27 levels showed a moderate albeit significant correlation with sPD-L1 concentration. Altogether, these data suggest a potential role of IL-27 in shaping PD-L1-mediated immune resistance in MM.

## Figures and Tables

**Table 1:** Association of sPD-L1 concentration with IL-27 and IL-6 concentrations.

	Total	sPD-L1 ≥ 166.2 pg/mL				
		N	%	OR *	95% CI ^¶^	*p*-Value
IL-27 < 0.358 ng/mL	38	12	30.8	1 (ref)	-	0.001
IL-27 ≥ 0.358 ng/mL	39	27	69.2	4.86	1.86–12.79	
IL-6 < 1683.5 pg/mL	39	20	51.3	1 (ref)	-	0.910
IL-6 ≥ 1683.5 pg/mL	38	19	48.7	0.950	0.39–2.32	

* OR, odds ratio. ^¶^ 95% CI, 95% confidence intervals.

## Data Availability

The data presented in this study are contained within the article and supplementary materials.

## Supplementary Materials

The following are available online at https://www.mdpi.com/article/10.3390/cancers13164011/s1, Figure S1: Western blot analysis of tyrosine phosphorylated (p)STAT1, pSTAT3, total STAT1 and total STAT3 proteins in MSTO and MPP89 cells stimulated for 20 min with medium (CTR), IL-27 or IFN-γ. Figure S2: (**A**) Both IL-27 and IFN-γ upregulate PD-L1 molecule expression by malignant mesothelioma (MM) cells. (**B**) IL-27 upregulates MHC Class I molecule expression by MM cells. Figure S3: Analysis of IFN-γ levels in MM pleural exudates. Figure S4: Densitometric analyses of relevant bands (panels 4.1 to 4.3) and whole Western blot images (panels 4.4 to 4.12), relative to the figures indicated in each panel. Table S1: qRT-PCR primers. Table S2: Patient characteristics.
