# Supplementary material for: IL-27 Mediates PD-L1 Expression and Release by Human Mesothelioma Cells
Source: Cancers (Basel). 2021 Aug 9;13(16):4011. doi: 10.3390/cancers13164011 (PMC8393193; doi:10.3390/cancers13164011)
Supplement: Supplementary file 1 [file cancers-13-04011-s001.zip › cancers-1303293_supplementary.pdf]

Carbotti G. et al. IL-27 mediates PD-L1 expression and release by human mesothelioma cells

SUPPLEMENTARY MATERIAL

SUPPLEMENTARY FIGURE S1

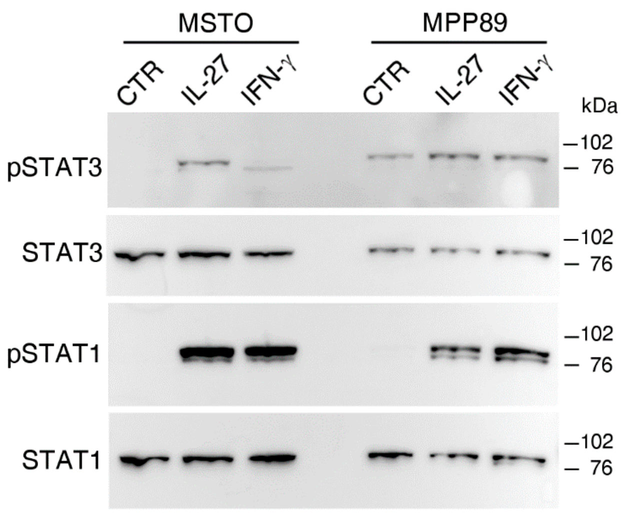

Supplementary Figure S1. Western blot analysis of tyrosine phosphorylated (p)STAT1, pSTAT3, total STAT1 and total STAT3 proteins in MSTO and MPP89 cells stimulated for 20 minutes with medium (CTR), IL-27 or IFN-γ. Total STATs were used as loading controls.

Stimulation with IFN-γ predominantly induces STAT1 phosphorylation in MM cell lines in a similar manner as IL-27.

SUPPLEMENTARY FIGURE S2

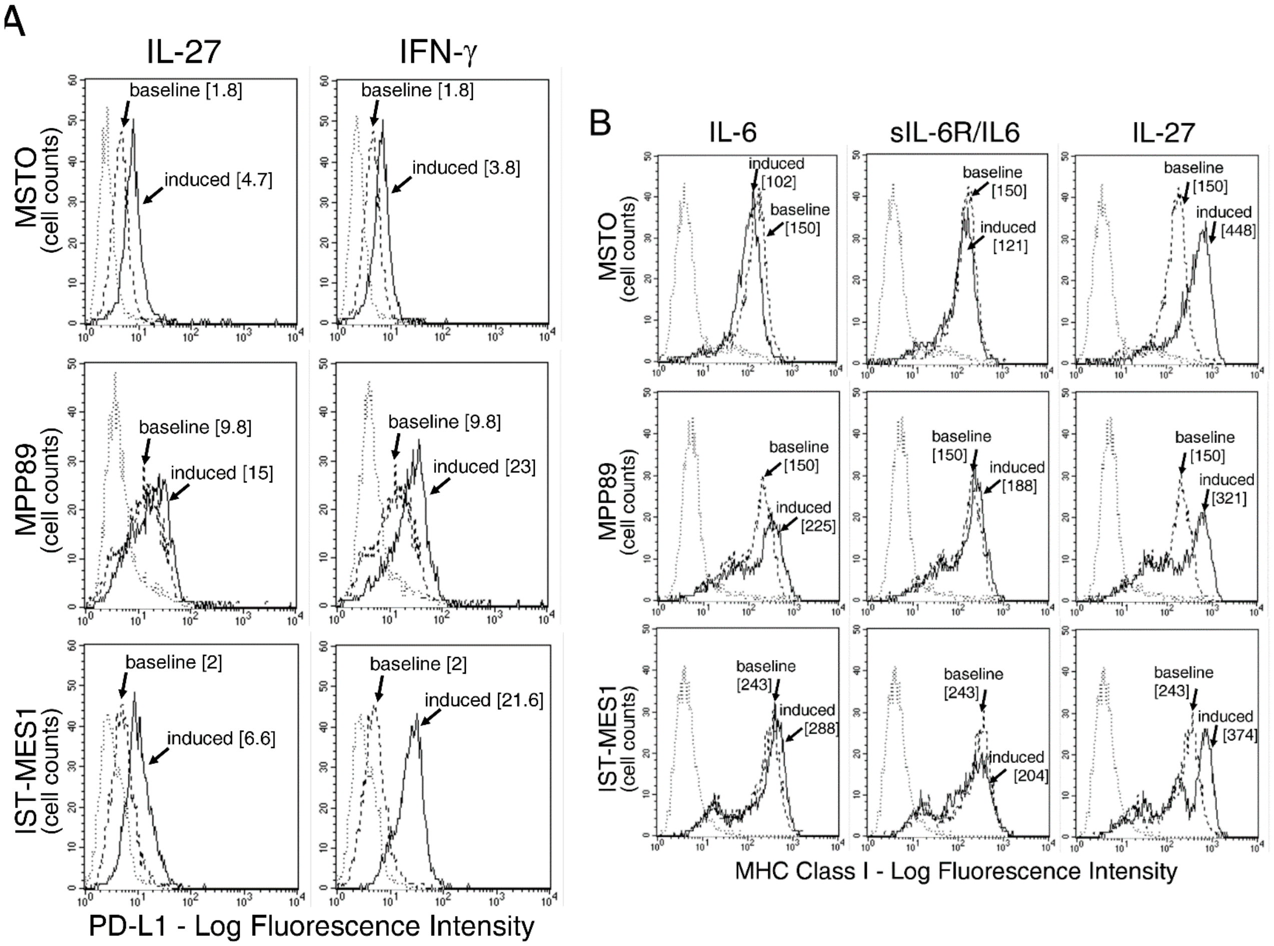

Supplementary Figure S2. Both IL-27 and IFN-γ upregulate PD-L1 molecule expression by malignant mesothelioma (MM) cells and IL-27 upregulates MHC Class I molecule expression by MM cells. (**A**) Flow cytometry analysis of membrane PD-L1 expression in MSTO, MPP89 and IST-MES1 cell lines cultured with medium alone (baseline), IL-27 or IFN-γ (induced). Dotted line shows isotype-matched Ig control. Numbers in brackets represent Median Fluorescence Intensity (MFI) values calculated as median anti-PD-L1 mAb minus median Ig control. Data are representative of two independent experiments yielding similar results. (**B**) Flow cytometry analysis of membrane MHC Class I expression in MSTO, MPP89 and IST-MES1 MM cell lines cultured with medium alone (baseline), IL-6, sIL-6R/IL-6 chimera or IL-27 (induced). Dotted lines show isotype-matched Ig control. Numbers in brackets represent Median Fluorescence Intensity (MFI) values calculated as

median anti-MHC Class I W6/32 mAb minus median Ig control. Data are representative of two independent experiments yielding similar results.

SUPPLEMENTARY FIGURE S3

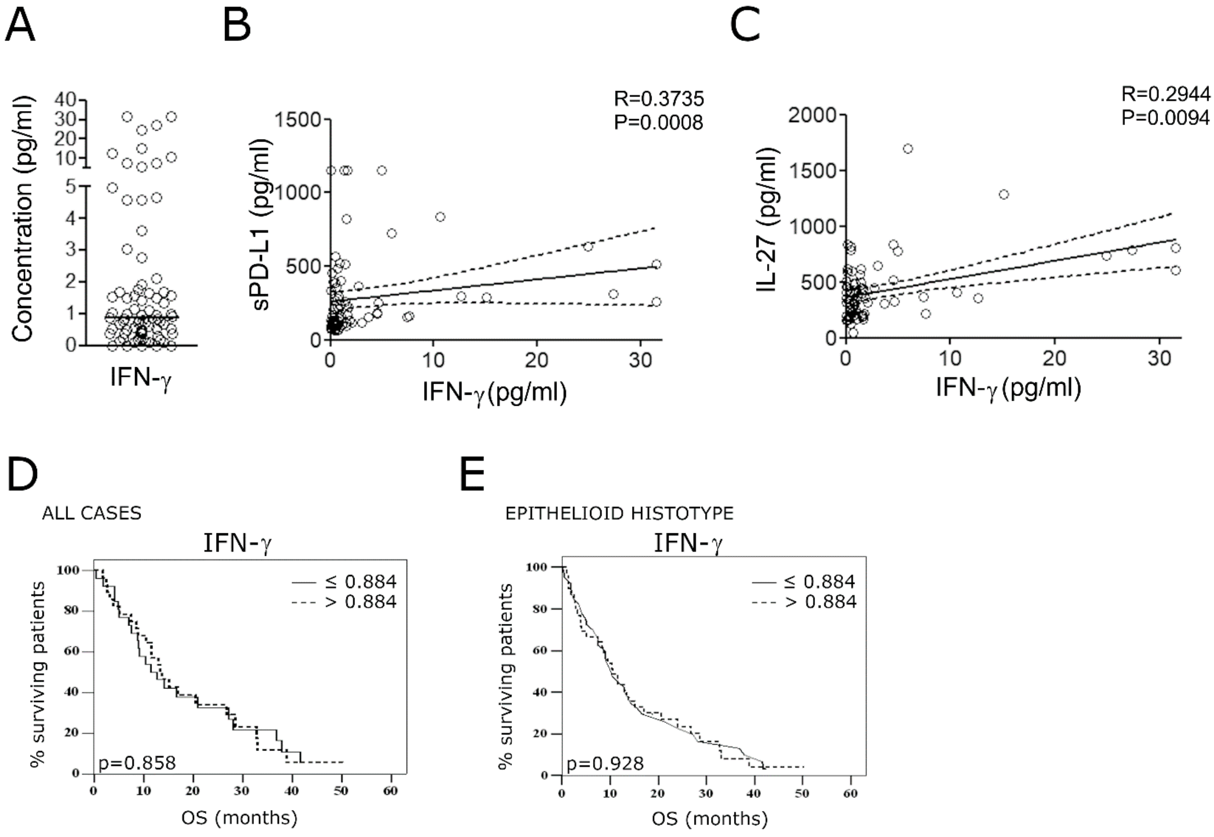

Supplementary Figure S3. Analysis of IFN-γ levels in MM pleural exudates. Samples were re-assessed by a high sensitivity ELISA assay (Quantikine HS ELISA, code HSDIF0, R&D Systems, Minneapolis, MN). With this assay, the values obtained were all within the standard curve range, thus making the results reliable for statistical evaluation. (A) Concentration (pg/ml) of IFN-γ in pleural effusion. Bar indicates median value. (B) Correlation between IFN-γ and sPD-L1 levels in MM pleural fluids. (C) Correlation between IFN-γ and IL-27 in the same cohort. Spearman's rank correlation coefficient (R) and p value (P) are indicated. Lines represent the best fit linear regression analysis with the 95% confidence interval. (D, E) Kaplan-Meier curves assessing overall survival according to IFN-γ

concentrations in the pleural effusion, as dichotomized around the median value. Analyses with respect to all cases (n=77) (D) and the subgroup with the epithelioid histotype (n=55) (E) are shown.

SUPPLEMENTARY FIGURE S4

Densitometric analyses of relevant bands in Western blot

4.1

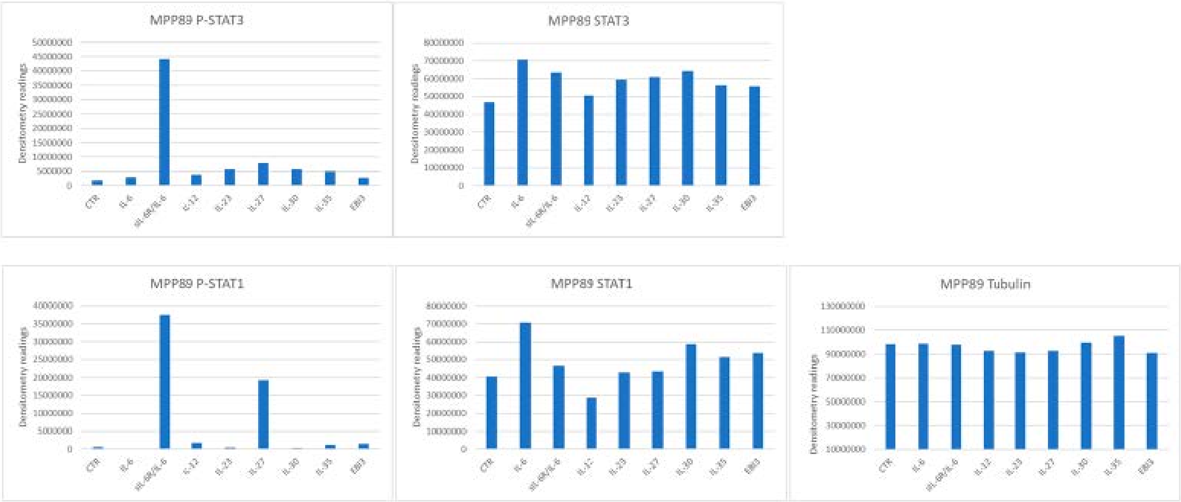

Densitometric analyses of relevant bands in western blot

4.2

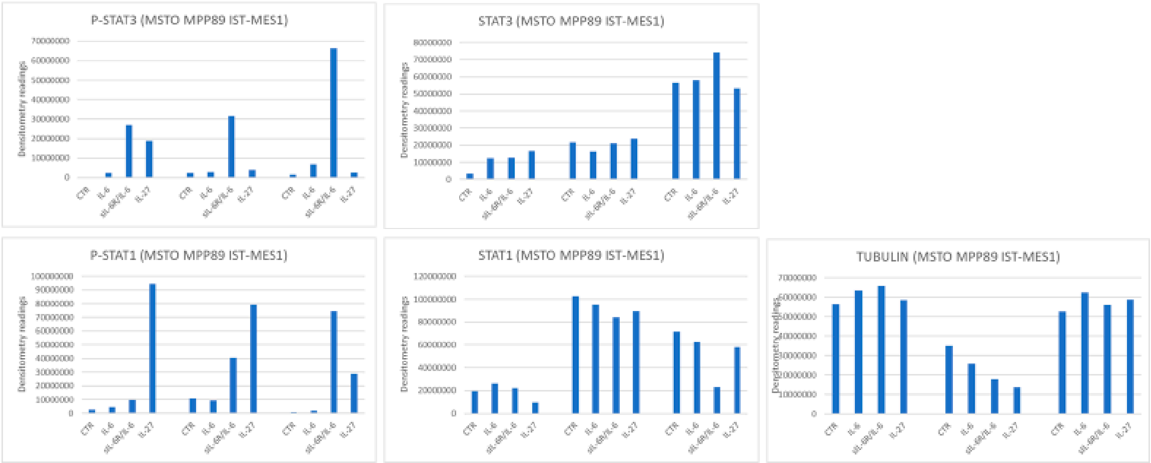

Densitometric analyses of relevant bands in western blot

4.3

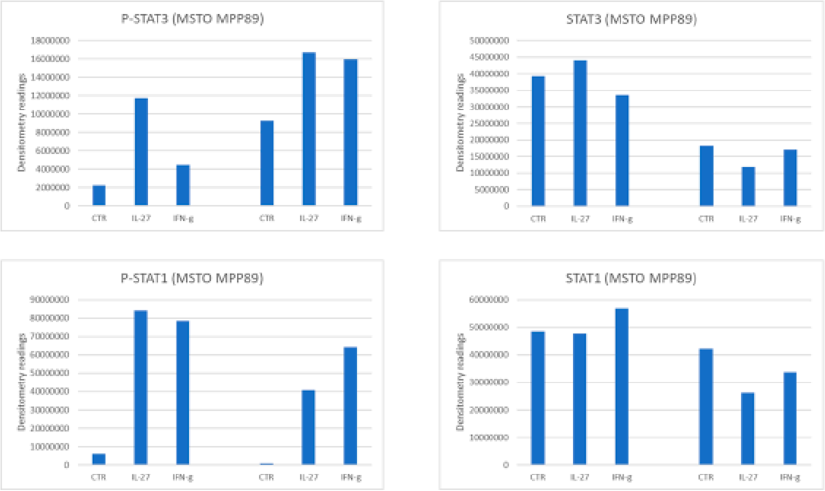

Whole Western blot images, cropped in figure: 4.4

MPP89

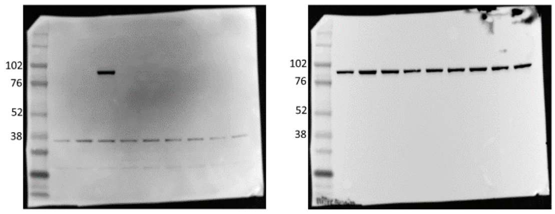

Probe: anti-phospho-STAT3

Probe: anti-STAT3

Carbotti G et al. Whole Western blot images, cropped in figure: 4.5

MPP89

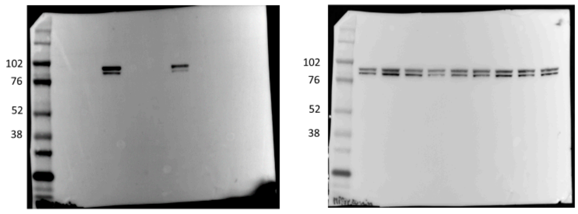

Probe: anti-phospho-STAT1

Probe: anti-STAT1

Markers: GE RPN800E

Carbotti G et al. Whole Western blot images, cropped in figure: 4.6

MPP89

MSTO and MPP89

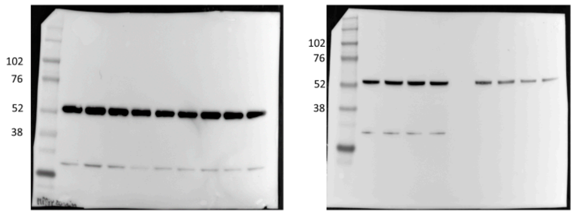

Probe: anti-Tubulin

Probe: anti-Tubulin

Carbotti G et al. Whole Western blot images, cropped in figure: 4.7

MSTO and MPP89

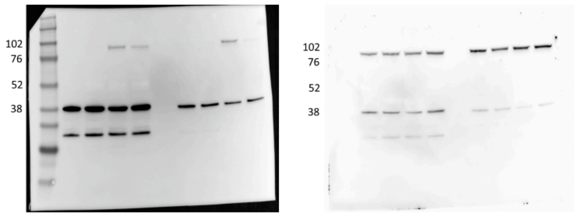

Probe: anti-phospho-STAT3

Probe: anti-STAT3

Carbotti G et al. Whole Western blot images, cropped in figure: 4.8

MSTO and MPP89

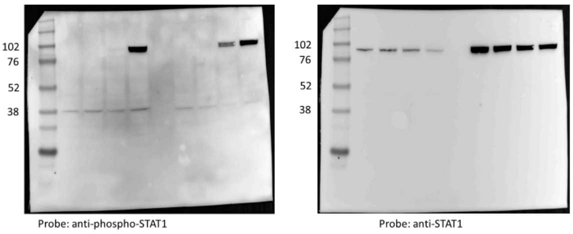

Carbotti G et al. Whole Western blot images, cropped in figure: 4.9

IST-MES1

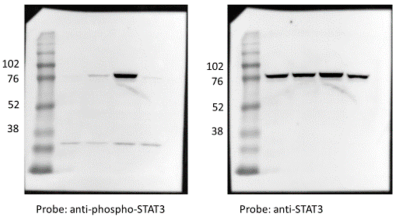

Carbotti G et al. Whole Western blot images, cropped in figure: 4.10

IST-MES1

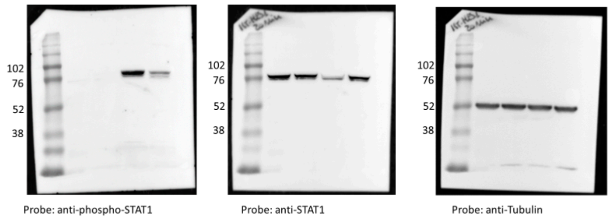

Carbotti G et al. Whole Western blot images, cropped in figure: 4.11

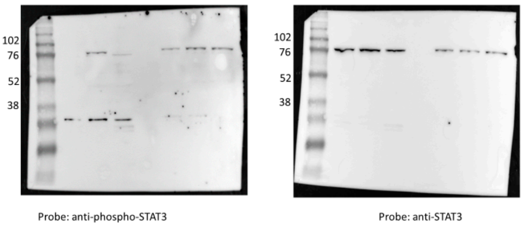

Carbotti G et al. Whole Western blot images, cropped in figure: 4.12

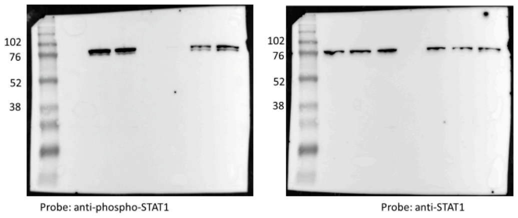

Supplementary Figure S4. Densitometric analyses of relevant bands (panels 4.1 to 4.3) and whole Western blot images (panels 4.4 to 4.12), relative to the figures indicated in each panel.

SUPPLEMENTARY TABLE S1. **qRT-PCR primers**.

Gene	Forward primer	Reverse primer
GAPDH	GAAGGTGAAGGTCGGAGT	CATGGGTGGAATCATATTGGAA
POLR2A	GACAATGCAGAGAAGCTGG	GCAGGAAGACATCATCATCC
CD274 (PD-L1)	TGCCGACTACAAGCGAATTACTG	CTGCTTGTCCAGATGACTTCGG
LGALS3 (GAL3)	CCATCTTCTGGACAGCCAAGTG	TATCAGCATGCGAGGCACCACT
GBP1	TAGCAGACTTCTGTTCCTACATCT	CCACTGCTGATGGCATTGACGT

SUPPLEMENTARY TABLE S2. Patient characteristics. The cohort (n=77) was obtained from the "Alessandria Biobank-Centro Raccolta Materiali Biologici", Department of Integrated Activities Research and Innovation, Azienda Ospedaliera SS. Antonio e Biagio e Cesare Arrigo, Alessandria, Italy.

	n (%)
Age (around median)	
≤74y	39 (50.6)
>74y	38 (49.4)
Gender	
Male	56 (72.7)
Female	21 (27.3)
Histology	
Epithelioid	55 (71.4)
Sarcomatoid	12 (15.6)
Biphasic	10 (13.0)
Stage	
I	1 (1.3)
II	17 (22.0)
III-IV	35 (45.5)
Unknown	24 (31.2)
ECOG	
0	11 (14.3)
1	6 (7.8)
≥2	5 (6.5)
Unknown	55 (71.4)
Exposure to asbestos	
Environmental	29 (37.7)
Professional	29 (37.7)
Unknown	19 (24.6)
Smoking history	
Never smoked	28 (36.4)

Current smoker	5 (6.5)
Ex-smoker	25 (32.4)
Unknown	19 (24.7)
Therapy	
No	22 (28.6)
At least 1 line	48 (62.3)
Unknown	7 (9.1)
